# Irradiation and conditioned media from human umbilical cord stem cells suppress epithelial-mesenchymal transition biomarkers in breast cancer cells

**DOI:** 10.22038/IJBMS.2023.68374.14919

**Published:** 2023-04

**Authors:** Rahil Ghanbarnasab Behbahani, Amir Danyaei, Hamed Shogi, Mohammad Javad Tahmasbi, Ghasem Saki, Niloofar Neisi

**Affiliations:** 1 Department of Medical Physics, School of Medicine, Ahvaz Jundishapur University of Medical Sciences, Ahvaz, Iran; 2 Department of Anatomical Sciences, School of Medicine, Ahvaz Jundishapur University of Medical Sciences, Ahvaz, Iran; 3 Department of Virology, School of Medicine, Ahvaz Jundishapur University of Medical Sciences, Ahvaz, Iran

**Keywords:** Breast carcinoma, EMT markers, MDA-MB-231 cells, Mesenchymal stem cell, Radiotherapy

## Abstract

**Objective(s):**

Breast cancer cells developing radioresistance during radiation may result in cancer recurrence and poor survival. One of the main reasons for this problem is the changes in the regulation of genes that have a key role in the epithelial-mesenchymal transition (EMT). Utilizing mesenchymal stem cells can be an effective approach to overcome therapeutic resistance. In this study, we investigated the possibility of combining mesenchymal medium with cancer cell medium in sensitizing breast carcinoma cells to radiation.

**Materials and Methods:**

In this experimental study, the cells were irradiated at a dose of 4 Gy alone and in combination with stem cells and cancer cells media. Apoptosis, cell cycle, Western blotting, and real-time PCR assays evaluated the therapeutic effects.

**Results:**

We found that the CSCM could decrease the expression of several EMT markers (CD133, CD44, Vimentin, Nanog, Snail, and Twist), resulting in increased cell distribution in the G1 and G2/M phases, apoptosis rate, and protein levels of p-Chk2 and cyclin D1; furthermore, it exhibits synergetic effects with radiation treatment *in vitro*.

**Conclusion:**

These findings show that CSCM inhibits the expansion of breast cancer cells and makes them more susceptible to radiotherapy, offering a unique approach to treating breast cancer by overcoming radioresistance.

## Introduction

Breast cancer is the world’s most prevalent cancer nowadays, despite having early detection programs in countries since the 1980s (1). Depending on the type of breast cancer and how advanced it is, it might need radiotherapy, chemotherapy, and hormone therapy either before or after surgery, or sometimes both (2). Nonetheless, its curative efficacy is sometimes limited by resistance responses, recurrence, and metastasis (3). Therefore, it is essential to look for new therapeutic approaches to concretely target tumor cells and increase patients’ overall survival.

Mesenchymal stem cells (MSCs) are one of the efficient strategies in clinical medicine. The MSC’s potential ability to self-renew and differentiate into a wide range of cell types results in their application in the treatment of diverse pathologies, including neurological disorders, cardiac ischemia, and diabetes (4, 5). Because of the ability to differentiate and transform into other cells, stem cells are used to repair damaged tissues (6). The accessible sources of stem cells include bone marrow, umbilical cord, adipose tissue, and placenta (3). Numerous reports have indicated that MSCs act as a double-edged sword because they can either promote or suppress tumor behavior under various conditions (7, 8).

Due to the lack of known molecular receptors in triple-negative breast cancer (TNBC) cells and the decline in the patient’s overall survival in TNBC, it seems necessary to find molecular pathways and introduce new therapeutic approaches (1).

We investigated the synergistic effect of the MSCs medium and irradiation on MDA-MB-231 cells. Also, induction of apoptosis, cell cycle, gene expression, and protein expression have been examined to evaluate the response of cells to this combination therapy.

## Materials and Methods

The experimental groups were used for either non-irradiated or irradiated conditions (Listed in Table 1). The experimental techniques were performed 24 hr after irradiation. 

***Cell culture***

The human breast cancer cell line MDA-MB-231 (IBRC) was grown in high glucose DMEM medium supplemented with 10% fetal bovine serum (FBS, BIO-IDEA-Iran), 1% nonessential amino acid (Sigma, USA), and 1% penicillin-streptomycin (BIO-IDEA-Iran) at 37  ^°^C and 5% CO_2_. In order to grow and multiply the human umbilical cord MSCs, after separating the blood vessels and the amniotic membrane from the umbilical cord, it was washed three times with phosphate-buffered saline (PBS) containing antibiotics. The human umbilical cord MSCs were provided by the Noor Genetics Lab (Ahvaz- Iran). The human umbilical cord MSCs were cultured in DMEM/F12 (BIO-IDEA-Iran), 20% FBS, and 1% penicillin-streptomycin (BIO-IDEA-Iran). The expression of cell surface markers CD105, CD90, CD34, and CD45 was examined by FACSCalibur flow cytometer (BD Diagnostics, Franklin Lakes, NJ) to confirm the cells were mesenchymal (2).

***Collection of conditioned medium***

The supernatants of MDA-MB-231 and MSC cultures were collected after reaching 70-90% confluence and stored at -80 ^°^C.

***Cell irradiation***

The cells were centered in the irradiation chamber and irradiated using a single dose of 4 Gy of 6 MV photons (Varian, Golestan General Hospital, Ahvaz, Iran).

***Cell morphology***

The breast cancer cell line was seeded in a 6-well plate. The medium was replaced with the appropriate medium for the CSCM and Msc groups the next day. Changes in the appearance of cells were observed by an inverted microscope (Hund, Wetzlar, Germany). 

***Apoptosis assay***

In order to detect the induction of apoptosis, the MDA-MB-231 cells were seeded in 6-well plates. After 24 hr of irradiation, the cells were digested by trypsin and washed twice with cold PBS and binding buffer. Then, the cells were resuspended in 500 μl of binding buffer and stained with 5 μl Annexin V-FITC and 5 μl PI. Finally, the cells were incubated for 15 min in darkness and analyzed using a flow cytometer (BD FACSCalibur, USA).

***Cell cycle analysis***

First, the cells of treatment groups were harvested and washed with PBS and fixed for at least 30 min at 4 in cold 70% ethanol. Then the cells were washed and treated with RNase A. Afterward, PI was added, and the cells were incubated for 15 min. Finally, the fluorescence intensity was analyzed by a flow cytometer (BD FACSCalibur, USA).

***Western blot analysis***

Western blotting was executed to recognize the particular protein in MDA-MB-231 cells. After examining different treatments, the protein was extracted using RIPA lysis buffer containing protease inhibitor cocktails (Melford, UK) on ice. The total protein was quantiﬁed using the Bradford assay. The samples were loaded on a 10% SDS-PAGE (Bio-Rad) and were transferred to a nitrocellulose membrane (membrane solution; Shanghai, China). Then the membranes were blocked in the 5% skim milk in TBST buffer overnight at 4 ^°^C. After washing membranes with TBST five times, they were incubated with primary antibodies (pChk2 (Thr 68), cyclin D1, and β-actin (Santa Cruz, USA) at 4 ^°^C overnight. The next day, washing the membranes with TBST five more times, they were blotted with a secondary antibody (anti-rabbit IgG, Santa Cruz, USA) for 1 hr at room temperature. Finally, the protein bands were developed using Clarity™ Western ECL Substrate (Bio-Rad, USA) detection reagent and visualized using the ChemiDoc MP system (Bio-Rad). Protein levels were quantified by ImageJ software.

***Real-time polymerase chain reaction (PCR) technique***

Total RNA was extracted from the cells using RNX Plus reagent (SinaClon, Iran) according to instructions. Total RNA (3 µg) was reverse transcribed using a cDNA Kit (Qiagen, Germany), and real-time PCR was performed using the RealQ Plus 2x Master Mix Green (Denmark). The method was used to quantify the relative mRNA expression. The expression levels of genes (listed in Table 2) were normalized by *HPRT* as the internal reference.

***Statistical analysis***

The non-parametric Kolmogorov-Smirnov test assessed the normality of the data. Data were analyzed with one-way ANOVA using GraphPad Prism software. The tests were repeated three times to complete the results, which were presented as mean±SD, and the *P*-value below 0.05 was considered statistically significant in all experiments.

## Results

***Effect of CSCM medium combined with radiotherapy on cell morphology***

**Figure 1:**
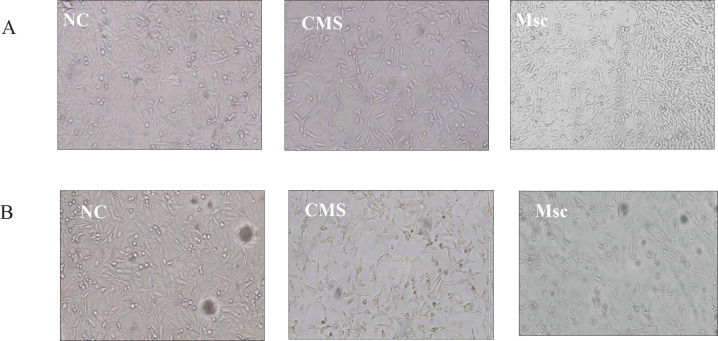
Morphology analysis of the cells indicated by an inverted microscope

Microscope analysis indicated that spindle cells appeared lengthy and slender, and the cell connections were reduced in treatment groups with the media of the MSCs. Irradiation had no effect on cell morphology in the NC group but caused a further decrease in the number of cells in the CSCM and Msc groups (Figure 1).

***CSCM medium combined with irradiation promoted apoptosis in the MDA-MB-231 cells***

**Figure 2:**
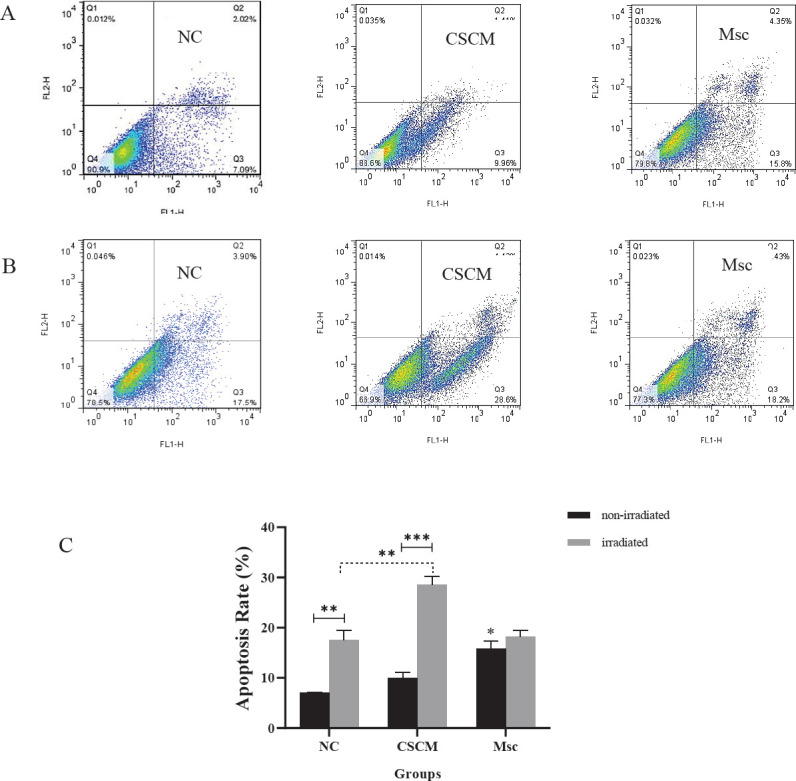
CSCM medium combined with irradiation promoted apoptosis in the MDA-MB-231 cells

Apoptosis assays by flow cytometry revealed that the rate of induced apoptosis in the Msc group was significantly increased compared with the NC group (*P*<0.05) before radiation. After irradiation, apoptosis induction was meaningfully increased in the CSCM group compared with the NC group (*P*<0.01). The results demonstrated that 4 Gy dose irradiation significantly increased the apoptosis rate in the NC (*P*<0.01) and the CSCM groups (*P*<0.001) in comparison with the non-irradiated same groups (Figure 2). 

***The CSCM medium combined with irradiation changed the cell cycle distribution***

**Figure 3:**
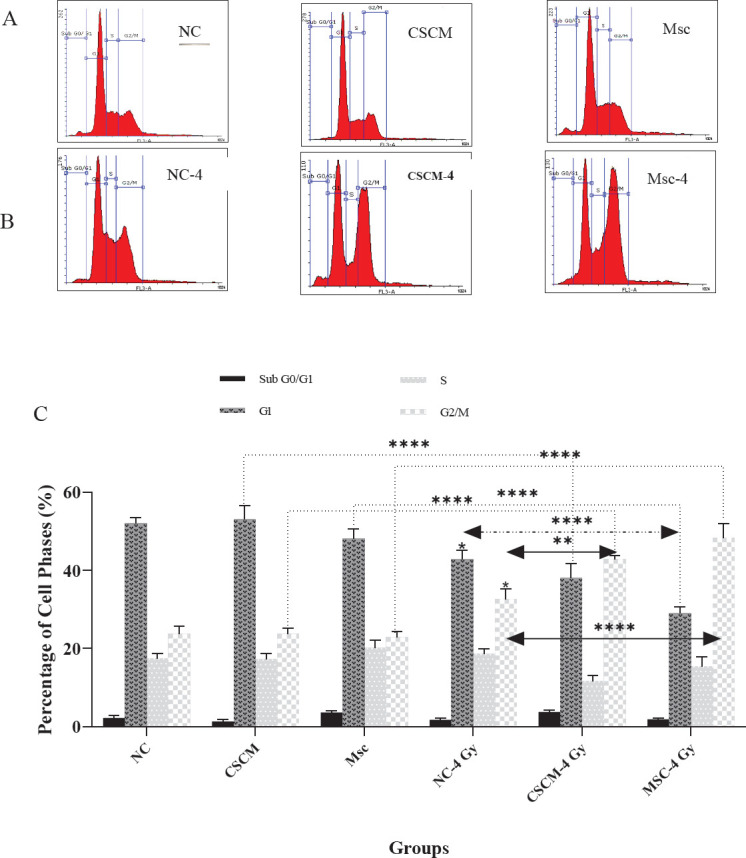
The CSCM medium combined with irradiation changed cell cycle distribution

After examining apoptosis to determine modifications in cell cycle distribution, we analyzed the treatment groups by flow cytometry. Comparing the cell percentages, we noticed no changes in different cell cycle phases before irradiation. After irradiation, the G1 and G2/M phases were significantly shortened, while they were lengthend in the NC group (*P*<0.05). Additionally, the proportion of cells in the G2/M phase was remarkably increased in the CSCM group compared with the NC group (*P*<0.01). Meanwhile, the number of cells in the G1 phase was meaningfully decreased in the Msc group compared with the NC group (*P*<0.0001). However, the proportion of cells in the G2/M phase showed significant growth (*P*<0.0001). Moreover, data analysis demonstrated a considerable decline in the percentage of cells located in the G1 phase. Whereas; the cell population of the G2/M phase was substantially increased in the Msc group compared with the non-irradiated corresponding group (*P*<0.0001 in both) (Figure 3).

***Effect of the CSCM medium combined with irradiation on proteins that alter cell cycle progression***

**Figure 4:**
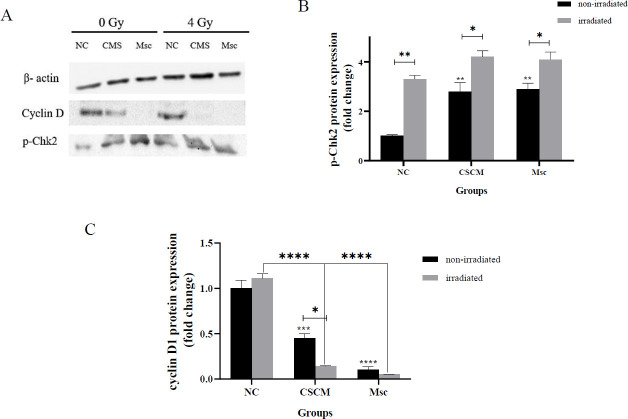
Effect of the CSCM medium combined with irradiation on proteins that alter cell cycle progression

To identify different proteins that alter cell cycle progression, we determined the p-Chk2 and cyclin D1 protein levels by the Western blotting technique. The data indicated that the MSC medium could enhance the p-Chk2 protein expression in the CSCM and Msc groups compared with the NC group (*P*<0.01 in both). To apply radiation of 4 Gy single dose, no changes in protein expression level in all treatment groups compared to each other. Nonetheless, the p-Chk2 protein level increased significantly in the CSCM and Msc groups compared with the respective non-irradiated group (*P*<0.05 in both). Furthermore, the investigation of cyclin D1 protein showed that its expression declined remarkably in the CSCM and Msc groups compared to the NC group (*P*<0.001 and* P*<0.0001, respectively). Also, radiation of 4 Gy single dose caused substantial decrements the expression of cyclin D1 in the CSCM and Msc groups compared with the NC group (P<0.0001 in both). The combination of MSC medium and irradiation together led to a significant reduction only in the CSCM group compared to the non-irradiated CSCM group (*P*<0.05) (Figure 4).

***Effect of the stem cells’ medium in combination with radiotherapy*** ***on the mRNA expression of epithelial-mesenchymal transition (EMT) markers***

**Figure 5:**
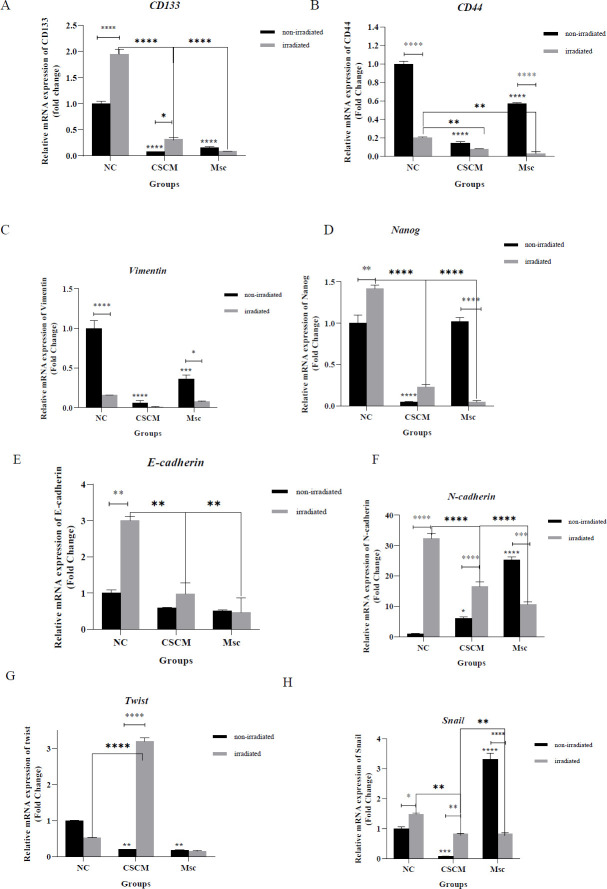
Effect of the stem cells’ medium in combination with radiotherapy on mRNA expression of Epithelial Mesenchymal Transition (EMT) markers. The diagrams show the expression of genes that were examined based on real-time qPCR

To investigate the effect of stem cells’ medium alone or along with irradiation, we appraised the mRNA expression of several EMT markers. We evaluated MSC surface- and structure-related markers like *CD133*, *CD44*, *N-cadherin*, and *Vimentin*; surface- and structure-related genes involved in EMT transcription such as *Snail*, *Twist*, and *Nanog* and an epithelial marker (*E‐Cadherin*). Before irradiation, mRNA levels of *CD133*, *CD44*, *Vimentin*, *Nanog*, *Twist*, and *Snail* in the CSCM were significantly lower than the NC group (*P*<0.0001,* P*<0.0001,* P<*0*.*0001,* P*<0.0001,* P*<0.01, and* P*<0.001, respectively). Also, the *N-cadherin* mRNA expression was significantly increased compared with the NC group (*P*<0.05). In the Msc group, *CD133*, *CD44*, *Vimentin*, and *Twist* were meaningfully decreased in comparison with the NC group (*P*<0*.*0001,* P*<0*.*0001,* P*<0*.*001, and* P*<0*.*01, respectively). At the same time, the *N-cadherin* and *Snail* were considerably enhanced compared with the NC group (*P*<0*.*0001* in both*). After irradiation, the mRNA expression of the *CD44* and *Vimentin* were remarkably reduced, and *CD133*, *Nanog*, *E-cadherin*, *N-cadherin*, and *Snail* were substantially declined in the NC group compared with the non-irradiated NC group (*P*<0*.*0001* in both*,* P*<0*.*0001,* P*<0*.*01,* P*<0*.*01,* P*<0*.*0001, and* P*<0*.*05, respectively). In the CSCM group, the mRNA levels of the *CD133*, *CD44*, *Nanog*, and *E-cadherin* were notably decreased compared with the NC group (*P*<0*.*0001,* P*<0*.*01,* P*<0*.*0001, and* P*<0*.*01, respectively). Meanwhile, mRNA expressions of the *Twist*, *N-cadherin*, and *Snail* showed a significant increase in comparison to the NC group (*P*<0*.*0001,* P*<0*.*0001, and* P*<0*.*01, respectively). In the Msc group, except for a significant increase in the *N-cadherin* expression, other markers, including the *CD133*, *CD44*, *Nanog*, and *E-cadherin* were remarkably declined compared with the NC group (*P*<0*.*0001,* P*<0*.*0001,* P*<0*.*01,* P*<0*.*0001, and* P*<0*.*01, respectively). The results illustrated a considerable increase in the *CD133*, *Twist*, *N-cadherin*, and *Snail* mRNAs and a significant reduction in the *Vimentin* in the CSCM group compared with the non-irradiated CSCM group (*P*<0*.*05,* P*<0*.*0001,* P*<0*.*0001, and* P*<0*.*01, respectively). Whereas the *CD44*, *Nanog*, *N-cadherin*, and *Snail* were meaningfully decreased in the Msc group related to the non-irradiated Msc group (*P*<0*.*0001,* P*<0*.*0001,* P*<0*.*001, and* P*<0*.*0001, respectively) (Figure 5). 

**Table 1:** The experimental groups of cells used in this study

Experimental Groups	Description
**NC**	The group with the medium of the MDA-MB-231 cells
CSCM	The group with the medium of the MDA-MB-231 cells and MSCs (1:1) **(cancer/stem cell medium)**
**Msc**	The group with the MSC medium

**Table 2:** The primer sequences used for the real-time quantitative polymerase chain reaction

**Gene Name**	**Forward Sequence (5'-3')**	**Reverse Sequence (5'-3')**
***E-cadherin***	CGAGAGCTACACGTTCACGG	GGGTGTCGAGGGAAAAATAGG
***CD44***	CATGGACAAGTTTTGGTGGCAC	GCAAAGCGGCAGGTTATATTC
***Vimentin***	ACCAGCTAACCAACGACAAAG	GACGCATTGTCAACATCCTGT
***CD133***	ACCAGCGACAGAAGGAAAATG	CTTGAAATTGCTATCTGCCAGTT
***Snail***	CCAGTGCCTCGACCACTATG	CTGCTGGAAGGTAAACTCTGGA
***Twist***	CTACGCCTTCTCGGTCTG	TGGAAACAATGACATCTAGGTCTC
***N-cadherin***	AGGCTTCTGGTGAAATCGCA	TGCAGTTGCTAAACTTCACATTG
***Nanog***	CAATGGTGTGACGCAGAAGG	GAAGGTTCCCAGTCGGGTTC
***HPRT***	TAGCCCTCTGTGTGCTCAAG	ACTTTTATGTCCCCTGTTGACTG

(A) The groups without irradiation and (B) the groups with irradiation

(A) Representative images of apoptosis in groups treated by the medium of MDA-MB-231 cells and MSC cells, and (B) combined with 4 Gy radiation. (C) The rate of apoptosis is shown as the mean±SD (error bars) from three independent experiments (* *P<*0.05, ** *P<*0.01, and *** *P<*0.001)

(A) Non-irradiated groups and (B) irradiated groups. (C) Quantitative evaluation of cell proportions at each phase (*P<0.05, ***P<0.001, and ****P<0.0001)

(A) The p-Chk2 and cyclin D1 proteins were identified using the Western blot assay and normalized with the beta-actin protein. (B) The p-Chk2 protein levels in different groups. (C) The cyclin D1 protein levels in different groups (*P<0.05, **P<0.01, ***P<0.001, and ****P<0.0001)

(A) the expression of the CD133 gene; (B) the expression of the CD44 gene; (C) the expression of the Vimentin gene; (D) the expression of the Nanog gene; (E) the expression of the *E-cadherin* gene; (F) the expression of the *N-cadherin* gene; (G) the expression of the Twist gene; (H) the expression of the Snail gene (* *P<*0.05, ** *P<*0.01, *** *P<*0.001, and****P<0.0001)

## Discussion

Nowadays, cancer treatment is facing several challenges. Numerous reasons reduce the efficiency of cancer therapy outcomes and even cause metastasis and recurrence in more advanced stages, including drug resistance, lack of epigenetic profile, deficiency of efficient biomarkers for cancer diagnosis and prognosis, and cancer stem cells (9). Furthermore, some studies have indicated that ionizing radiation could facilitate metastasis of invasive tumor cells through EMT; notwithstanding, radiotherapy is one of the main approaches to cancer therapy (10, 11). On the contrary, several studies have demonstrated that MSCs hold promising potential for cancer therapy (12, 13). Hence, researchers are endeavoring to find the most effective procedure to treat cancer. In this study, we applied the human Wharton’s jelly MSC medium combined with a dose of 4 Gy in order to increase radiosensitivity in the MDA-MB-231 breast carcinoma cell line. 

Numerous studies have shown that several factors are specific features of stem cells, such as Vimentin, Nanog, Twist, N-cadherin, E-cadherin, CD133, CD44, and Snail (14, 15). Low expression of E-cadherin and high expression of other markers can assist in maintaining mesenchymal characteristics of the cells. Furthermore, cancer stem cells lead to chemo-radiotherapy and recurrence in metastatic cancer cells. In terms of self-renewal and differentiation, cancer stem cells are identical to regular stem cells. They also contribute to cancer cell chemoresistance and metastasis, resulting in treatment failure (16). As previously stated, epithelial markers (E-cadherin) and mesenchymal markers (N-cadherin) are the two primary elements in stem cell analysis. The expression of these two markers is considered to be independent of one another. According to research, E-cadherin is required for epithelial cell adhesion and reduced motility. Due to diminished E-cadherin expression in tumor cells, cancer cells may be able to penetrate the basement membrane and invade other organs (17). Nevertheless, N-cadherin overexpression causes enhanced motility, invasion, and metastasis, even when E-cadherin, a tumor suppressor, is present (18). Kim and co-workers have shown that radiation increases N-cadherin expression in the MCF-7 cell line (19). In our study, using the CSCM medium alone or in combination with radiation did not affect enhancing *E-cadherin* and declining *N-cadherin* expression. However, 4G radiation promoted *E-cadherin* expression while decreasing *N-cadherin* expression when combined with a mesenchymal medium.Vimentin and Nanog are essential for the cytoskeleton of mesenchymal cells as well as the extension of metastasis via EMT, and their overexpression contributes to poor prognosis in breast carcinoma (14, 20). Inhibiting Nanog can reduce stemness and promote apoptosis, while its high expression in MDA-MB-231 cells leads to poor prognosis, invasion, and migration (21). The findings revealed that combining the medium of mesenchymal cells and cancer cells alone or with irradiation reduced their expression considerably. Other genes with elevated expression in breast cancer include *Snail* and *Twist*, which inhibit apoptosis, enhance angiogenesis, and cause chromosomal instability. Upregulation these genes, also reduced the level of E-cadherin, which facilitates the EMT process and growing CSCs (22-24). According to Lane *et al.*, radiation alone can promote the expression of Twist in TNBC cells (25). We found that combining mesenchymal and malignant medium may dramatically lower *Twist* and *Snail* expression, although combining this medium with radiation increases their expression. Following that, two cell surface markers, CD44 and CD133, were examined for further research. Some research has shown that *CD133* and *CD44* are used as markers to identify cancer stem cells in diverse cancers like breast carcinoma. Radiation resistance, migration, and invasion are all assisted through the high expression of these genes. A study has shown that cancer cells with CD133^+^ specificity have higher radioresistance and lower apoptosis than cancer cells with CD133^- ^(26, 27). 

We examined p-Chk2 and cyclin D1 to evaluate the effects of applying the mesenchymal medium and 4G radiation on the expression of DNA damage proteins and cell survival pathways, respectively. Most normal cells in the body express Cyclin D1, which regulates intracellular pathways and proliferation. On the other hand, impaired transcription and D1 accumulation might result in uncontrolled cell proliferation. As a result, it is classified as an oncogenic stimulant in a range of cancers, like breast neoplasm (28, 29). In addition, p-Chk2 activity signals DNA damage, which promotes the ATM and Chk2 proteins (30). Our results exhibited that the CSCM medium with radiation alone or together could increase the p-Chk2 and cyclin D1 protein levels.

## Conclusion

We used a combination of stem cells and a cancer cell culture media. Furthermore, cell sensitivity was investigated by applying a 4 Gy dose. Our data indicated that the CSCM had synergistic effects with radiation therapy *in vitro*, decreasing the expression of several EMT markers and increasing significant cell distribution in the G1 and G2/M phases, apoptosis rate, and protein levels of p-Chk2 and cyclin D1. 

## Authors’ Contributions

AD, GHS, and HSH contributed to conception and design. RGHB and HSH contributed to all experimental work, data and statistical analysis, and interpretation of data. MJT participated in the irradiation part and NN participated in the cultivation and analysis of apoptosis data. AD was responsible for overall supervision. RGHB prepared the draft of the manuscript, which was revised by AD. All authors read and approved the final manuscript.

## Conflicts of Interest

The authors declare that they have no conflicts of interest.

## References

[1] Chavez KJ, Garimella SV, Lipkowitz S (2010). Triple negative breast cancer cell lines: One tool in the search for better treatment of triple negative breast cancer. Breast Dis.

[2] Hashemitabar M, Allahbakhshi E, Tabande MR, Orazizadeh M, Dehbashi FN, Azandeh S (2015). Isolation and characterization of human umbilical cord mesenchymal stem cells and their differentiation into Pdx-1+ Cells. J Biomed Sci Eng.

[3] Gudkov AV, Komarova EA (2003). The role of p53 in determining sensitivity to radiotherapy. Nat Rev Cancer.

[4] Jia-Quan Q, Hong-Mei Y, Xu Y, Li-Na L, Jin-Feng Z, Ta X (2015). MiR-23a sensitizes nasopharyngeal carcinoma to irradiation by targeting IL-8/Stat3 pathway. Oncotarget.

[5] Brown JM, Wouters BG (1999). Apoptosis, p53, and tumor cell sensitivity to anticancer agents. Cancer Res.

[6] Wang X, Lin Y (2008). Tumor necrosis factor and cancer, buddies or foes?. Acta Pharmacol Sin.

[7] Zhang Z, Lin G, Yan Y, Li X, Hu Y, Wang J (2018). Transmembrane TNF-alpha promotes chemoresistance in breast cancer cells. Oncogene.

[8] Yu M, Zhou X, Niu L, Lin G, Huang J, Zhou W (2013). Targeting transmembrane TNF-alpha suppresses breast cancer growth. Cancer Res.

[9] Chakraborty S, Rahman T (2012). The difficulties in cancer treatment. Ecancermedicalscience.

[10] De Bacco F, Luraghi P, Medico E, Reato G, Girolami F, Perera T (2011). Induction of MET by ionizing radiation and its role in radioresistance and invasive growth of cancer. J Natl Cancer Inst.

[11] Wild-Bode C, Weller M, Rimner A, Dichgans J, Wick W (2001). Sublethal irradiation promotes migration and invasiveness of glioma cells: implications for radiotherapy of human glioblastoma. Cancer Res.

[12] Ramasamy R, Lam EW, Soeiro I, Tisato V, Bonnet D, Dazzi F (2007). Mesenchymal stem cells inhibit proliferation and apoptosis of tumor cells: Impact on in vivo tumor growth. Leukemia.

[13] Lin HD, Fong CY, Biswas A, Choolani M, Bongso A (2016). Human umbilical cord wharton’s jelly stem cell conditioned medium induces tumoricidal effects on lymphoma cells through hydrogen peroxide mediation. J Cell Biochem.

[14] Brabletz T, Kalluri R, Nieto MA, Weinberg RA (2018). EMT in cancer. Nat Rev Cancer.

[15] Lamouille S, Xu J, Derynck R (2014). Molecular mechanisms of epithelial-mesenchymal transition. Nat Rev Mol Cell Biol.

[16] Vasefifar P, Motafakkerazad R, Maleki LA, Najafi S, Ghrobaninezhad F, Najafzadeh B (2022). Nanog, as a key cancer stem cell marker in tumor progression. Gene.

[17] Weinberg RA, Weinberg RA (2006). The Biology of Cancer.

[18] Hazan RB, Phillips GR, Qiao RF, Norton L, Aaronson SA (2000). Exogenous expression of N-cadherin in breast cancer cells induces cell migration, invasion, and metastasis. J Cell Biol.

[19] Kim R-K, Kaushik N, Suh Y, Yoo K-C, Cui Y-H, Kim M-J (2016). Radiation driven epithelial-mesenchymal transition is mediated by Notch signaling in breast cancer. Oncotarget.

[20] Ulirsch J, Fan C, Knafl G, Wu MJ, Coleman B, Perou CM (2013). Vimentin DNA methylation predicts survival in breast cancer. Breast Cancer Res Treat.

[21] Serej ZA, Ebrahimi A, Kazemi T, Najafi S, Amini M, Nastarin P (2021). NANOG gene suppression and replacement of let-7 modulate the stemness, invasion, and apoptosis in breast cancer. Gene.

[22] Zhu QQ, Ma C, Wang Q, Song Y, Lv T (2016). The role of TWIST1 in epithelial-mesenchymal transition and cancers. Tumour Biol.

[23] Vesuna F, Bergman Y, Raman V (2017). Genomic pathways modulated by Twist in breast cancer. BMC cancer.

[24] Kurrey NK, Jalgaonkar SP, Joglekar AV, Ghanate AD, Chaskar PD, Doiphode RY (2009). Snail and slug mediate radioresistance and chemoresistance by antagonizing p53-mediated apoptosis and acquiring a stem-like phenotype in ovarian cancer cells. Stem Cells.

[25] Lin Y, Bai X, Zhou W, He Y, Wu Y, Wang X (2018). Radiation exposure triggers the progression of triple negative breast cancer via stabilizing ZEB1. Biomed Pharmacother.

[26] Chiou SH, Kao CL, Chen YW, Chien CS, Hung SC, Lo JF (2008). Identification of CD133-positive radioresistant cells in atypical teratoid/rhabdoid tumor. PLoS One.

[27] Ke CC, Liu RS, Yang AH, Liu CS, Chi CW, Tseng LM (2013). CD133-expressing thyroid cancer cells are undifferentiated, radioresistant and survive radioiodide therapy. Eur J Nucl Med Mol Imaging.

[28] He Y, Liu Z, Qiao C, Xu M, Yu J, Li G (2014). Expression and significance of Wnt signaling components and their target genes in breast carcinoma. Mol Med Rep.

[29] Arnold A, Papanikolaou A (2005). Cyclin D1 in breast cancer pathogenesis. J Clin Oncol.

[30] Yin H, Glass J (2011). The phenotypic radiation resistance of CD44+/CD24- or low breast cancer cells is mediated through the enhanced activation of ATM signaling. PLoS One.

